# The SRC family kinase inhibitor NXP900 demonstrates potent antitumor activity in squamous cell carcinomas

**DOI:** 10.1016/j.jbc.2024.107615

**Published:** 2024-07-31

**Authors:** Sweta Dash, Sabrina Hanson, Ben King, Katherine Nyswaner, Kelcie Foss, Noelle Tesi, Mungo J.B. Harvey, Saúl A. Navarro-Marchal, Allison Woods, Enrique Poradosu, Asier Unciti-Broceta, Neil O. Carragher, John Brognard

**Affiliations:** 1 Laboratory of Cell and Developmental Signaling, Center for Cancer Research, National Cancer Institute at Frederick, NIH, Frederick, Maryland, USA; 2 Edinburgh Cancer Research, Institute of Genetics and Cancer, University of Edinburgh, Edinburgh, UK; 3 Cancer Research UK Scotland Centre, Edinburgh, UK; 4 Nuvectis Pharma Inc, Fort Lee, New Jersey, USA

**Keywords:** Src family kinases, inhibitor, HNSCC, ESCC, anti-cancer drug

## Abstract

NXP900 is a selective and potent SRC family kinase (SFK) inhibitor, currently being dosed in a phase 1 clinical trial, that locks SRC in the “closed” conformation, thereby inhibiting both kinase-dependent catalytic activity and kinase-independent functions. In contrast, several multi-targeted kinase inhibitors that inhibit SRC, including dasatinib and bosutinib, bind their target in the active “open” conformation, allowing SRC and other SFKs to act as a scaffold to promote tumorigenesis through non-catalytic functions. NXP900 exhibits a unique target selectivity profile with sub-nanomolar activity against SFK members over other kinases. This results in highly potent and specific SFK pathway inhibition. Here, we demonstrate that esophageal squamous cell carcinomas and head and neck squamous cell carcinomas are exquisitely sensitive to NXP900 treatment in cell culture and *in vivo*, and we identify a patient population that could benefit from treatment with NXP900.

The SRC family of kinases (SFK) consists of nine non-receptor tyrosine (Tyr, Y) kinases that transfer the gamma phosphate of ATP to the hydroxyl residues of tyrosine in substrate proteins. In humans, the SFK members include SRC, YES, HCK, LCK, FYN, FGR, LYN, YRK, and BLK. These cytoplasmic tyrosine kinases regulate numerous normal cellular processes, including cell proliferation, adhesion, motility, differentiation, and survival (1).

Of the nine SFK members, SRC kinase has been extensively studied for decades since the discovery of the retrovirus-encoding gene *v-SRC*, a transmissible agent responsible for the development of tumors in chickens (2). The human homolog, encoded by proto-oncogene *c-SRC*, is regulated by the phosphorylation of tyrosine residues at 419 and 530. Phosphorylation at Y419 stabilizes the activation loop in the kinase domain permits substrate binding and enhances kinase activity, whereas phosphorylation at Y530 stabilizes a “closed,” inactive conformation that suppresses kinase activity (3). Under normal physiological conditions, SRC is tightly regulated by the C-terminal SRC kinase family members (such as CSK and CHK) that phosphorylate it at Y530, thereby inactivating it and serving as key negative regulators (4). Aberrant expression or activation of SRC has been observed in multiple cancer types, including squamous cell carcinomas, breast, prostate, pancreatic, and ovarian cancers, and often correlates with poor prognosis (5).

Current FDA-approved therapies that can inhibit SRC include ATP-competitive small-molecule inhibitors, such as dasatinib and bosutinib (6, 7). Both dasatinib and bosutinib are multi-kinase inhibitors that also target the nonreceptor tyrosine kinase ABL with equal potency. While dual inhibition of SRC and ABL is beneficial for the treatment of leukemia, wild-type ABL kinase has been shown to act as a tumor suppressor in many solid tumors (8, 9). In addition, inhibition of ABL leads to cardiotoxicity (10).

A novel, potent small-molecule SFK inhibitor, NXP900, was recently developed to target and stabilize SRC kinase in its “closed,” inactive conformation (11). This contrasts with the previously mentioned SRC/ABL inhibitors, including dasatinib, which bind to the active conformation of the SRC kinase (Fig. 1*A*). In addition, NXP900 displayed low single-digit nanomolar activity against SRC kinase, at concentrations three orders of magnitude lower than those required to inhibit ABL, indicating that it is highly selective to SRC compared with ABL (11). Several studies have shown that aberrant activation of SRC or other SFK members promotes tumorigenesis in esophageal (ESCC), head and neck (HNSCC) and lung squamous cell carcinomas (LSCC) (5, 12, 13). This study focuses on identifying cancer types, particularly among squamous cell carcinomas, that are sensitive to NXP900 treatment to identify a patient population that can benefit from treatment with NXP900.

**Figure 1:**
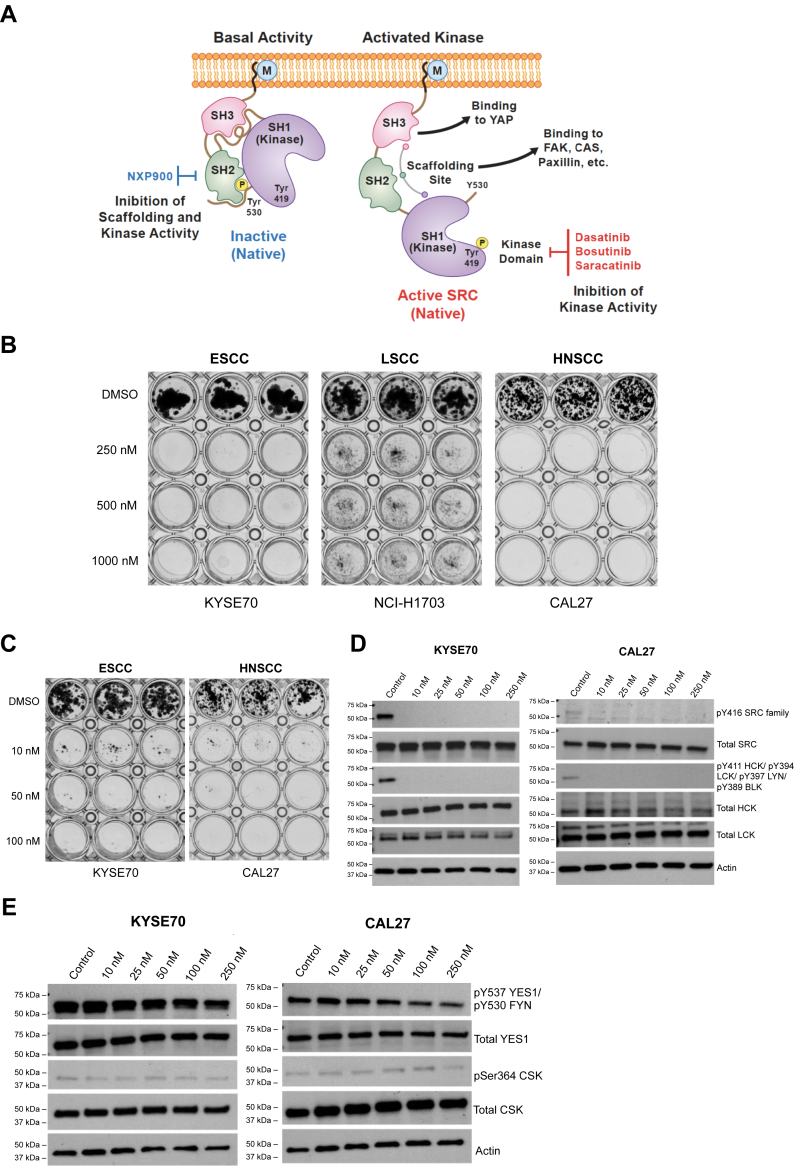
**SFK inhibition by NXP900.***A*, NXP900 locks SRC in its inactive conformation. *B and C*, initial screen of squamous cell carcinoma types showed that ESCC cell line KYSE70 and HNSCC cell line CAL27 had a significant reduction in cell viability in response to (*B*) high and (*C*) low concentrations of NXP900 in long-term (14-days) colony forming assay. *D*, activating phosphorylation levels of SRC family kinases were significantly reduced after 24 h of treatment with a low concentration of NXP900 in KYSE70 and CAL27 cells. *E*, no significant difference in inhibitory phosphorylation levels of YES1/FYN and activation levels of negative SRC regulator, CSK, was observed after NXP900 treatment.

## Results

### KYSE70 and CAL27 cell lines are sensitive to NXP900

We initially screened a cell line representing ESCC, HNSCC, and LSCC for sensitivity to NXP900. Long-term, 14-days treatment with varying concentrations of NXP900 demonstrated that the LSCC cell line NCI-H1703 had a residual resistant population of cells, even at a high concentration (1000 nM) of NXP900 (Fig. 1*B*). ImageJ quantification revealed ∼25% viable cells after 14 days of treatment with 1000 nM NXP900 in the NCI-H1703 cell line (Fig. S1*A*). In contrast, the ESCC cell line KYSE70 and the HNSCC cell line CAL27 were sensitive to NXP900 at concentrations as low as 10 nM (Figs. 1, *B* and *C* and S1*B*). No significant reduction in cell viability was observed at 1 nM and 5 nM concentrations of NXP900 in the CAL27 cell line (Fig. S1*C*).

We then assessed whether the reduction in cell viability in KYSE70 and CAL27 cell lines after NXP900 treatment was due to SFK inhibition. Immunoblotting revealed that NXP900 reduced activating phosphorylation levels of SRC (pY419) in KYSE70 (100%) and CAL27 (70%) after 24 h of treatment, starting at 10 nM (Figs. 1*D* and S1, *D*–*F*). In addition, treatment with 10 nM NXP900 completely reduced activating phosphorylation levels of other SFK members, such as LCK, HCK, LYN, and BLK (Figs. 1*D* and S1, *D*–*F*). In contrast, NXP900 treatment had no effect on the total kinase levels of SFK members (Figs. 1*D* and S1, *D*–*F*). Additionally, the negative regulatory phosphorylation site of SFK members YES1 (pY537) and FYN (pY530) remained unaffected by NXP900 treatment at concentrations up to 250 nM (Figs. 1*E* and S1, *D*–*F*). This is consistent with the mode of binding of NXP900, which blocks SFKs in their inactive conformation. The activating phosphorylation levels of the negative SFK regulator, CSK, were also unaffected after NXP900 treatment (Figs. 1*E* and S1, *D*–*F*), which further shows the high selectivity of the inhibitor. Kinome-wide activity profiling of 1 μM NXP900 demonstrated that only 25 out of 340 kinases had decreased activity below 50% (11). These results suggest that NXP900 specifically targets and inhibits SFK members.

### Reduction in cell viability in ESCC and HNSCC cell line panel in response to short-term and long-term NXP900 treatment

We observed that KYSE70 and CAL27 cell lines were highly sensitive to NXP900; therefore, we selected a panel of cell lines representing ESCC and HNSCC cancer types. For ESCC, we included the cell lines KYSE70, KYSE410, KYSE30, TE5, TE14, OE19, OE21 and OVCAR5, and for HNSCC, we evaluated cell viability in the BICR56, BICR6, BICR22, PECAPJ49, MSK921, FADU, CAL33 and CAL27 cell lines. These 16 cell lines were selected based on variable expression levels of *SRC* and *YES1* mRNA transcripts. The DepMap database was used for this selection process, with the x-axis denoting *SRC* transcript levels and the y-axis denoting *YES1* transcript levels (Fig. S2, *A* and *B*). Next, we performed immunoblotting to assess the SFK members’ protein expression, activation, and inhibition levels (Fig. S2, *C* and *D*). All ESCC and HNSCC cell lines displayed varying activation and expression levels of SFK members and CSK (Fig. S2, *C* and *D*).

**Figure 2:**
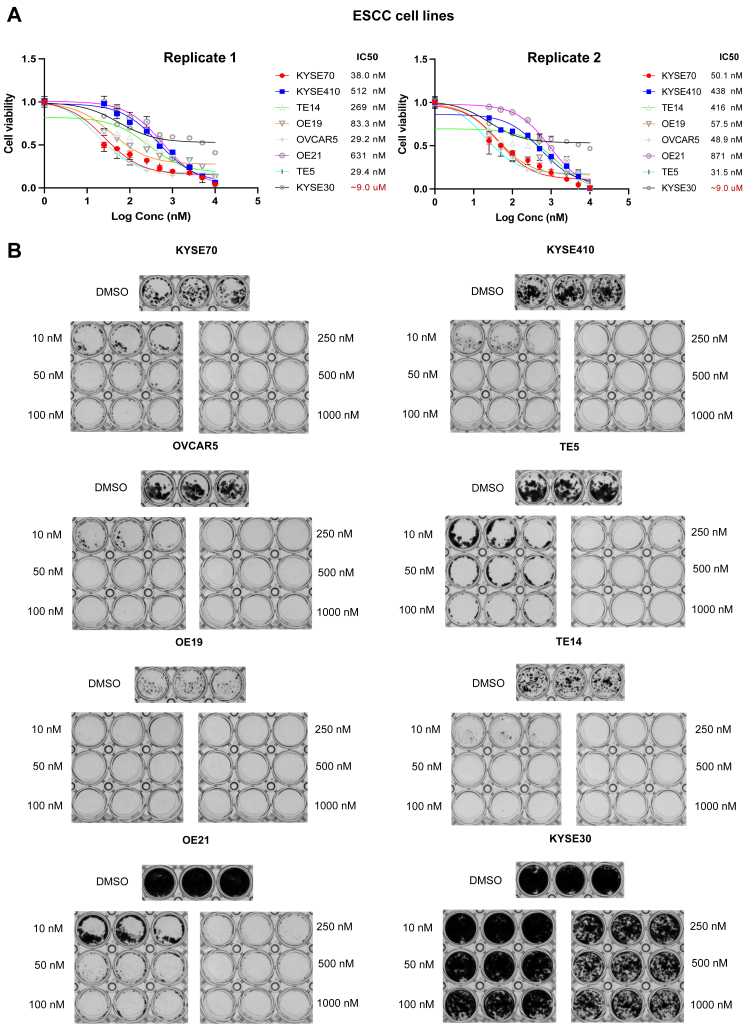
**Effect of NXP900 on ESCC cell viability.***A*, short-term treatment of ESCC cells with NXP900 for 72 h showed substantial reduction in cell viability, except for the KYSE30 cell line. *B*, long-term treatment of ESCC cells with NXP900 every other day for 14 days showed a significant reduction in cell viability at low concentrations, except for the KYSE30 cell line that showed the presence of a resistant population of cells, even at high concentrations of NXP900.

**Figure 3:**
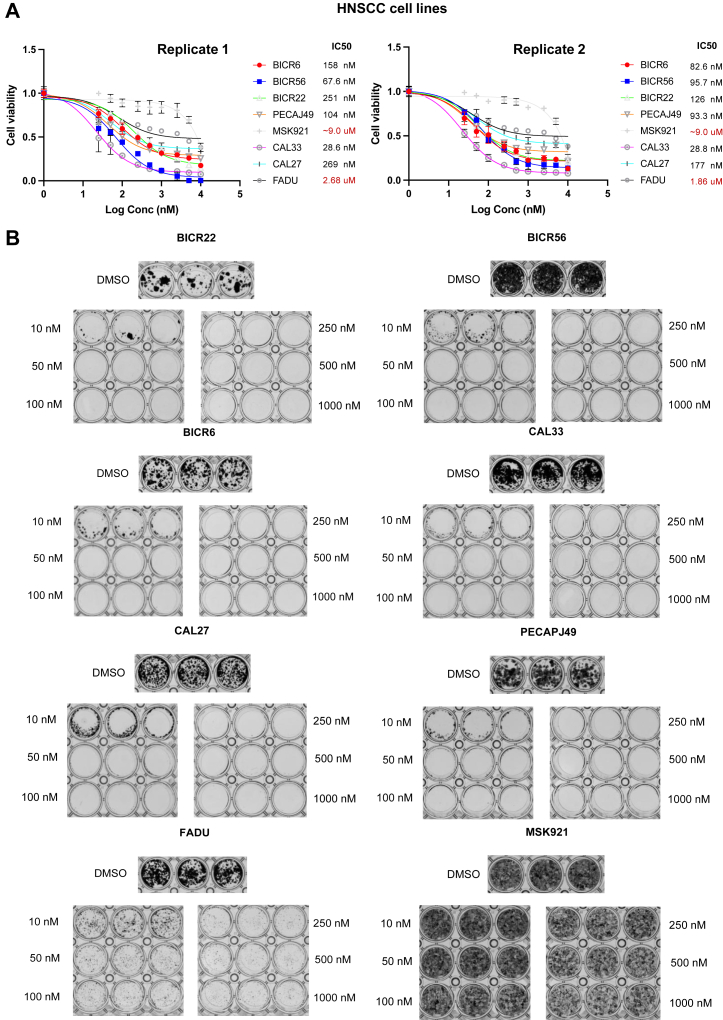
**Effect of NXP900 on HNSCC cell viability.***A*, short-term treatment of HNSCC cells with NXP900 for 72 h showed a substantial reduction in cell viability, except for FADU and MSK921 cell lines. *B*, long-term treatment of HNSCC cells with NXP900 every other day for 14 days showed a significant reduction in cell viability at low concentrations, except for FADU and MSK921 cell lines that showed the presence of a resistant population of cells, even at 1 uM NXP900.

Short-term, 72-h treatment with NXP900 revealed that ESCC cell lines, except for KYSE30, were sensitive to NXP900 (Fig. 2*A*). Similarly, six HNSCC cell lines showed a substantial reduction in cell viability after treatment with NXP900, while the FADU and MSK921 cell lines showed a slight reduction (Fig. 3*A*). In addition, a dose-response curve using Growth Rate (GR) metrics (14) was generated for all 16 cell lines using an online GR calculator to measure the effect of NXP900 treatment after normalizing for growth rate variations in different cell lines (Fig. S3, *A* and *B*). KYSE30, FADU, and MSK921 had higher GRmax and GR50 values (Fig. S3, *C* and *D*). A similar trend was observed after long-term, 14-day treatment with NXP900 in all 16 cell lines (Figs. 2*B* and 3*B*). The three cell lines that were least sensitive to NXP900 in the short-term MTS assay (KYSE30, FADU, and MSK921) also showed the presence of a resistant population of cells in the colony formation assays, even after long-term treatment with high concentrations of NXP900 at 1000 nM (Figs. 2*B* and 3*B*). Overall, our data indicate that ESCC and HNSCC cell lines are sensitive to NXP900 treatment by targeting the SRC family of kinases.

### NXP900 treatment reduced KYSE70 and CAL27 xenograft tumors in mouse models

**Figure 4:**
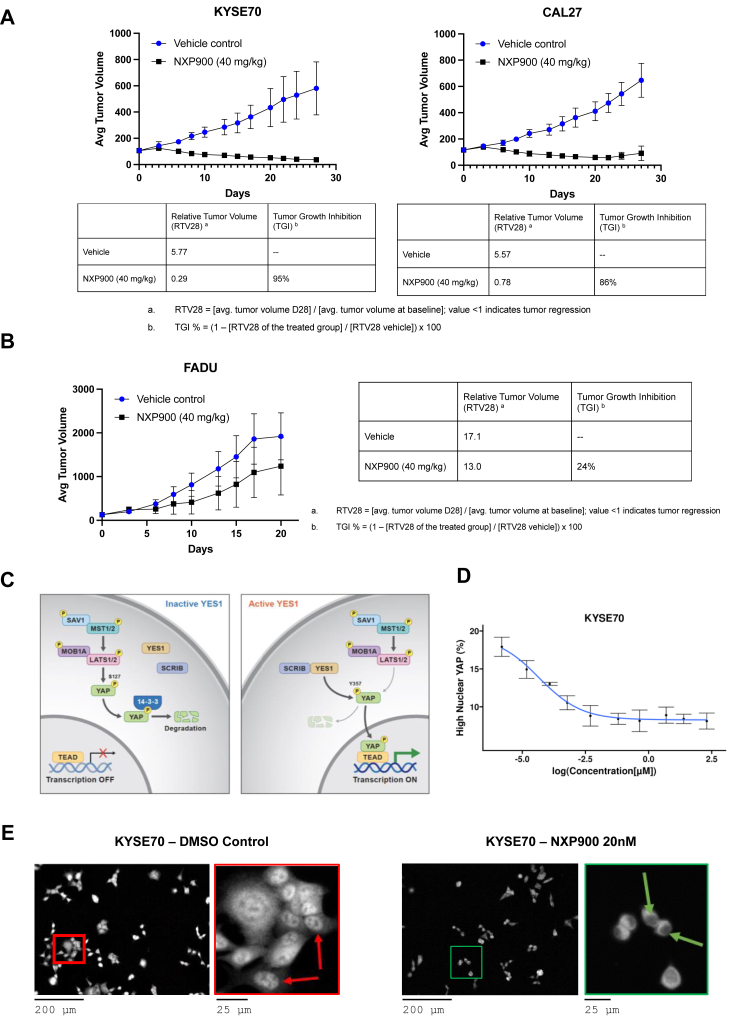
***In vivo* efficacy and YAP1 regulation.** Average tumour volumes significantly decreased when mice with (*A*) KYSE70 and CAL27 xenografts were treated with 40 mg/kg NXP900. *B*, mice with a FADU cell line xenograft did not show a significant decrease in tumour volume compared to control when treated with NXP900. *C*, schematic diagram of YAP1 regulation by YES1. *D*, dose-dependent reduction in high nuclear YAP in the KYSE70 cell line by NXP900 after 24 h of treatment. *E*, representative images of high (*red*) and low (*green*) nuclear YAP in the KYSE70 cell line.

To assess the efficacy of NXP900 *in vivo*, xenograft tumors were generated by subcutaneously implanting KYSE70, CAL27, or FADU cells in the right lower flank of the thigh of CD1 nude mice. After 28 days of treatment with 40 mg/kg NXP900, average tumor volumes were substantially reduced in KYSE70-and CAL27-xenografted mice compared to mice treated with vehicle control (Fig. 4*A*). In contrast, mice xenografted with the FADU cell line did not show a significant reduction in tumor volume after NXP900 treatment compared to vehicle control (Fig. 4*B*). This was consistent with the *in vitro* short-term and long-term effects of NXP900 treatment on KYSE70, CAL27, and FADU cell lines. These data highlight the potential of NXP900 as a new therapeutic intervention strategy for patients with ESCC and HNSCC.

### NXP900 treatment demonstrates dose-dependent inhibition of YAP1 nuclear localization

Previous studies demonstrate that SFK activity crosstalks with the Hippo signaling pathway, promoting stabilization and nuclear localization of the core Hippo pathway effector molecule, Yes-associated protein (YAP), to promote tumor progression (Fig. 4*C*) (15). Using automated high-content imaging and image analysis, we demonstrated that NXP900 treatment of KYSE70 cells resulted in a dose-dependent reduction of cells with high nuclear YAP expression (Fig. 4, *D* and *E*).

### High expression of TEAD2 correlates with NXP900 sensitivity in ESCC

We performed a bioinformatics analysis to determine whether any specific patterns of mutations, deletions, copy-number amplifications, and mRNA expression levels in known Hippo pathway modulators (from the DepMap database) correlate with NXP900 sensitivity across our ESCC panel. While no significant correlation between YAP1 mRNA expression levels with NXP900 sensitivity was observed (Fig. S4, *A* and *C*), higher expression levels of the downstream transcription factor of the Hippo pathway, TEAD2, strongly correlated with NXP900 sensitivity across the ESCC panel, while expression of two other Hippo mediators, AJUBA and DCHS1, showed a clear but non-significant trend (Fig. S4, *B* and *D*). Overall, these data will guide patient selection in the phase 2 clinical trial for NXP900.

## Discussion

Dual targeting of ABL and SRC using ATP-competitive small-molecule inhibitors, such as dasatinib and bosutinib, has emerged as a promising anti-tumor therapy in hematological malignancies. However, in solid tumors, these drugs have demonstrated limited efficacy (8, 10) due to drug resistance that can be mediated paradoxically by ATP-competitive inhibitors that trap SRC in the “open” conformation. Higuchi *et al.* demonstrated that inhibitor binding changes SRC’s conformation, causing SRC to associate with focal adhesion kinase (FAK), and can lead to ERK activation (16). The recently developed SRC/YES1 inhibitor, NXP900, potently targets inactive SRC/YES1, thereby preventing paradoxical activation of kinase-independent downstream signaling pathways (11).

In this study, we demonstrated that 13 ESCC and HNSCC cell lines were sensitive to NXP900 treatment in cell-based assays. Reduction in cell viability after long-term, 14-day treatment in cell-based assays was observed at low concentrations of NXP900 (10 nM). Additionally, we demonstrated that NXP900 reduced the activating phosphorylation levels of multiple SFK members, including SRC, HCK, LCK, LYN, and BLK at 10 nM concentration after 24 h of treatment. In contrast, NXP900 did not affect the inhibitory phosphorylation levels of SFK members YES1 and FYN1, in agreement with its inhibition mode of stabilizing the inactive conformation of SFKs.

NXP900 is also relevant to the Hippo signaling pathway. The pathway, composed of an upstream serine/threonine kinase signaling module and downstream transcriptional module, is highly conserved among species and is an important regulator of cell and organ growth. Deregulation of the Hippo pathway is found across a diverse range of cancers, where it has been suggested to be involved in cancer initiation and progression (17). The core effectors of the Hippo pathway are YAP1 and transcriptional co-activator with a PDZ-binding motif (TAZ), which bind to the transcription factor TEAD to induce gene expression. YAP and TAZ activity is principally controlled by these kinases’ cellular localization, where nuclear translocation enhances their ability to bind transcription factors such as TEAD. Several studies indicate that SFK activity can regulate YAP activity and nuclear accumulation through multiple mechanisms, including direct phosphorylation and modulation of upstream pathways repressing Hippo kinases (15). Next-generation sequencing studies demonstrate that modulation of mediators of the Hippo signaling pathway is relatively common across HNSCC and ESCC tumors correlating with poor patient prognosis (18, 19). Our own bioinformatics analysis demonstrated that mutations, deletions, and copy-number amplifications of several Hippo pathway modulators are present in cancer cell lines associated with high sensitivity to NXP900. Furthermore, NXP900 treatment resulted in a potent dose-dependent reduction of nuclear YAP in ESCC cells with high YAP expression. In addition, we showed that high TEAD2 expression levels strongly correlated with NXP900 sensitivity across the ESCC panel.

*In vivo* mouse xenograft experiments revealed that NXP900 treatment significantly reduced tumor volumes of KYSE70 and CAL27 cell line xenografts compared to vehicle control. NXP900 is currently in Phase 1 clinical trial, and our study demonstrates that ESCC and HNSCC patients with Hippo pathway modulations will be responsive to NXP900 and should be a patient cohort that is explored for treatment with NXP900.

## Experimental procedures

### Cell lines

KYSE70, KYSE30, KYSE410, TE5, TE14, OVCAR5, OE21, OE19, and MSK921 cell lines were cultured in RPMI media (Quality Biological) containing 10% FBS (Atlanta Biologicals) and 1× GlutaMax (Gibco). CAL27, CAL33, BICR56, BICR6, FADU, BICR22, and PECAPJ49 cell lines were cultured in DMEM (Sigma) containing 10% FBS, 0.4 μg/ml hydrocortisone and 1× GlutaMax. Cell lines tested negative for *mycoplasma* (*Mycoplasma* PCR Detection Kit, Applied Biological Materials, G238).

### Immunoblotting

Cells were seeded into 6-well plates at a density of 3 × 10^5^ cells/well, incubated overnight, and treated for 24 h with DMSO (vehicle control) and varying concentrations of NXP900: 10 nM, 25 nM, 50 nM, 100 nM, and 250 nM in fresh media. Whole-cell extracts were prepared by lysing the cells on ice in RIPA lysis buffer with protease and phosphatase inhibitors. The lysates were incubated on ice for 30 min, then centrifuged at 4 °C and 15,000 rpm for 18 min. Protein concentrations were determined with Pierce 660 nm Protein Assay Reagent. Lysates containing equivalent amounts of protein were run through sodium dodecyl sulfate-polyacrylamide gel electrophoresis (SDS-PAGE) in 4 to 20% gradient gels (Bio-Rad) and transferred into polyvinylidene fluoride (PVDF) membranes. Western blots were analyzed with pY416 SRC Family, Total SRC, pY411 HCK/pY394 LCK/pY397 LYN/pY389 BLK, Total HCK, Total LCK, pY537 YES1/pY530 FYN, Total YES1, pSER364 CSK, Total CSK, and actin. ImageJ software was used for quantification of the western blots.

### MTS assay

Two independent replicates of cells treated with NXP900 and DMSO (vehicle control) were performed. For each independent biological replicate, there were three technical replicates (total n = 6). Cells were seeded at a density of 2000 cells per well in 96-well plates. After overnight incubation in a 37 °C CO_2_ incubator, the cells were treated with DMSO (vehicle control) and varying concentrations of NXP900 (up to 10 uM) in triplicates. Then, 48 h after the first treatment, media were removed, and the cells were treated for the second time with NXP900 in fresh media. After overnight incubation, 10% v/v of MTS reagent (Promega) was added, and the plates were read at 490 nm using a microplate reader to measure the absorbance. Analysis was performed using GraphPad Prism (RRID:SCR_002798) software, and the absolute IC_50_ was calculated using “log(inhibitor) vs. response (four parameters)” method. Analysis for GR metrics was performed using an online GR calculator (http://www.grcalculator.org/grcalculator/). Cells were seeded at density of 2000 cells per well and incubated overnight in a 37 °C CO_2_ incubator. The following day cells were treated with 10% v/v of MTS reagent to get the initial cell count for the GR metrics. Input files were formatted using the ‘Case A (Recommended) - multiple cell counts per row’ format recommended by the GR calculator software. Standard error was selected in ‘Plot Options’ for the GR dose-response curves. GR50 and GRmax were selected in the ‘GR Metric Comparison’ section.

### Colony forming assay

Two independent replicates of cells treated with NXP900 and DMSO (vehicle control) were performed. For each independent biological replicate, there were three technical replicates (total n = 6). Cells were seeded in 24-well plates at a density of 1000 cells per well. The cells were then treated on alternate days with DMSO (vehicle control) and 10 nM, 50 nM, 100 nM, 250 nM, 500 nM, and 1000 nM of NXP900 in triplicates for 14 days. Colonies of cells were subsequently fixed in formaldehyde for 15 min. After formaldehyde fixation, 0.5% crystal violet solution (Sigma) was added to the plates, which were then incubated for 1 h. Plates were rinsed with distilled H_2_O and dried overnight. Images were taken using the BioRad ChemiDoc Imaging System.

### YAP subcellular localization assay

KYSE70 cells were seeded into 384-well plates and allowed to adhere for 24 h before treatment. Cells were treated with NXP900 in a 10-point dose-response ranging from 10 μM to 0.003 μM using the D300e Digital Dispenser (Tecan), incorporating three repeats per compound concentration. After 24 h of drug treatment, the cells were fixed with 4% formaldehyde for 20 min, washed with PBS, and then blocked and permeabilized for 30 min (0.3% Triton X-100, 1% BSA in PBS). Cells were labeled with YAP (1:500) overnight at 4 °C. Plates were washed in PBS again before being incubated with Hoechst (2 μg/ml), Phalloidin-iFluor 594 (1:1000) and Alexa-Fluor 488 (1:500) for 1 h at room temperature. The plates were subsequently washed again with PBS and then imaged using the ImageXpress Confocal high-content screening platform (Molecular Devices) to automatically acquire six images per well with a 20× objective lens. Images were automatically quantified using IN Carta software (Molecular Devices). The PhenoGlyphs module within IN Carta (RRID:SCR_000525) was used to classify cells as having high or low nuclear YAP: this method initially performed unbiased hierarchical clustering to identify cells of similar phenotypes, and we manually inspected and labeled the cells as having high or low nuclear YAP expression. These labels were used to iteratively train and improve a machine-learning classifier, which was then applied to all wells within each plate. Final data analysis was performed using R and tidyverse packages (20) to calculate the total percentage of high-nuclear-YAP cells within each well before we calculated dose-response metrics using the drc package (21). Data were visualized *via* ggplot2 (RRID:SCR_014601) by using the ggprism theme https://csdawgithubio/ggprism/, https://githubcom/csdaw/ggprism.

### Animal studies

Xenograft tumors were generated by subcutaneous implantation of cell lines (KYSE70, CAL27, and FADU) on the right lower flank of the thigh of CD1 nude mice at a cell density of 2 × 10^6^ to 1 × 10^7^ cells/mouse at 0.1 ml Matrigel/cell dilution volume per injection. Mice were treated with vehicle (citrate buffer 3 mM) and NXP900 (40 mg/kg) orally every day for 28 days Eight mice were used per cell line xenograft. Animal studies were approved by the CRO BRI Biopharmaceutical Research, Inc.

## Data availability

Authors declare that all data supporting the findings of this study is provided in the manuscript.

Note: NXP900 phase 1 clinical trial information - ClinicalTrials.gov Identifier: NCT05873686 (https://classic.clinicaltrials.gov/ct2/show/NCT05873686).

## Supporting information

This article contains supporting information.

## Conflict of interest

The authors declare the following financial interests/personal relationships which may be considered as potential competing interests:

AUB and NOC have received research grant funding from Nuvectis Pharma; in addition, AUB and NOC had patents to EP3298015B1, JP6684831B2, US10294227B2, CN107849050B, and CA3021550A1 licensed to Nuvectis Pharma.

## References

[1] Garmendia I., Redin E., Montuenga L.M., Calvo A. (2022). YES1: a novel therapeutic target and biomarker in cancer. Mol. Cancer Ther..

[2] Irby R.B., Yeatman T.J. (2000). Role of Src expression and activation in human cancer. Oncogene.

[3] Irtegun S., Wood R.J., Ormsby A.R., Mulhern T.D., Hatters D.M. (2013). Tyrosine 416 is phosphorylated in the closed, repressed conformation of c-Src. PLoS One.

[4] Zhu S., Wang H., Ranjan K., Zhang D. (2023). Regulation, targets and functions of CSK. Front. Cell Dev. Biol..

[5] Martellucci S., Clementi L., Sabetta S., Mattei V., Botta L., Angelucci A. (2020). Src family kinases as therapeutic targets in advanced solid tumors: what we have learned so far. Cancers.

[6] Marcos-Jiménez A., Carvoeiro D.C., Ruef N., Cuesta-Mateos C., Roy-Vallejo E., Gómez-García de Soria V. (2023). Dasatinib-induced spleen contraction leads to transient lymphocytosis. Blood Adv..

[7] Steinbach A., Clark S.M., Clemmons A.B. (2013). Bosutinib: a novel src/abl kinase inhibitor for chronic myelogenous leukemia. J. Adv. Pract. Oncol..

[8] Dasgupta Y., Koptyra M., Hoser G., Kantekure K., Roy D., Gornicka B. (2016). Normal ABL1 is a tumor suppressor and therapeutic target in human and mouse leukemias expressing oncogenic ABL1 kinases. Blood.

[9] Testoni E., Stephenson N.L., Torres-Ayuso P., Marusiak A.A., Trotter E.W., Hudson A. (2016). Somatically mutated ABL1 is an actionable and essential NSCLC survival gene EMBO. Mol. Med..

[10] Kerkelä R., Grazette L., Yacobi R., Iliescu C., Patten R., Beahm C. (2006). Cardiotoxicity of the cancer therapeutic agent imatinib mesylate. Nat. Med..

[11] Temps C., Lietha D., Webb E.R., Li X.F., Dawson J.C., Muir M. (2021). A conformation selective mode of inhibiting SRC improves drug efficacy and tolerability. Cancer Res..

[12] Chen J.Y., Hung C.C., Huang K.L., Chen Y.T., Liu S.Y., Chiang W.F. (2008). Src family kinases mediate betel quid-induced oral cancer cell motility and could be a biomarker for early invasion in oral squamous cell carcinoma. Neoplasia.

[13] Zhang J., Zhao D., Zhang L., Xiao Y., Wu Q., Wang Y. (2023). Src heterodimerically activates Lyn or Fyn to serve as targets for the diagnosis and treatment of esophageal squamous cell carcinoma. Sci. China Life Sci..

[14] Hafner M., Niepel M., Chung M., Sorger P.K. (2016). Growth rate inhibition metrics correct for confounders in measuring sensitivity to cancer drugs. Nat. Methods.

[15] Hsu P.C., Yang C.T., Jablons D.M., You L. (2020). The crosstalk between src and Hippo/YAP signaling pathways in non-small cell lung cancer (NSCLC). Cancers (Basel).

[16] Higuchi M., Ishiyama K., Maruoka M., Kanamori R., Takaori-Kondo A., Watanabe N. (2021). Paradoxical activation of c-Src as a drug-resistant mechanism. Cell Rep..

[17] Moroishi T., Hansen C.G., Guan K.L. (2015). The emerging roles of YAP and TAZ in cancer. Nat. Rev. Cancer.

[18] Faraji F., Ramirez S.I., Anguiano Quiroz P.Y., Mendez-Molina A.N., Gutkind J.S. (2022). Genomic hippo pathway alterations and persistent YAP/TAZ activation: new hallmarks in head and neck cancer. Cells.

[19] Gao Y.B., Chen Z.L., Li J.G., Hu X.D., Shi X.J., Sun Z.M. (2014). Genetic landscape of esophageal squamous cell carcinoma. Nat. Genet..

[20] Wickham H., Averick M., Bryan J., Chang W., McGowan L.D.A., François R. (2019). Welcome to the tidyverse. J. Open Source Softw..

[21] Ritz C., Baty F., Streibig J.C., Gerhard D. (2015). Dose-response analysis using R. PLoS One.

