# Supplementary material for: The SRC family kinase inhibitor NXP900 demonstrates potent antitumor activity in squamous cell carcinomas
Source: J Biol Chem. 2024 Jul 31;300(9):107615. doi: 10.1016/j.jbc.2024.107615 (PMC11388391; doi:10.1016/j.jbc.2024.107615)
Supplement: Supplementary Figures [file mmc1.pdf]

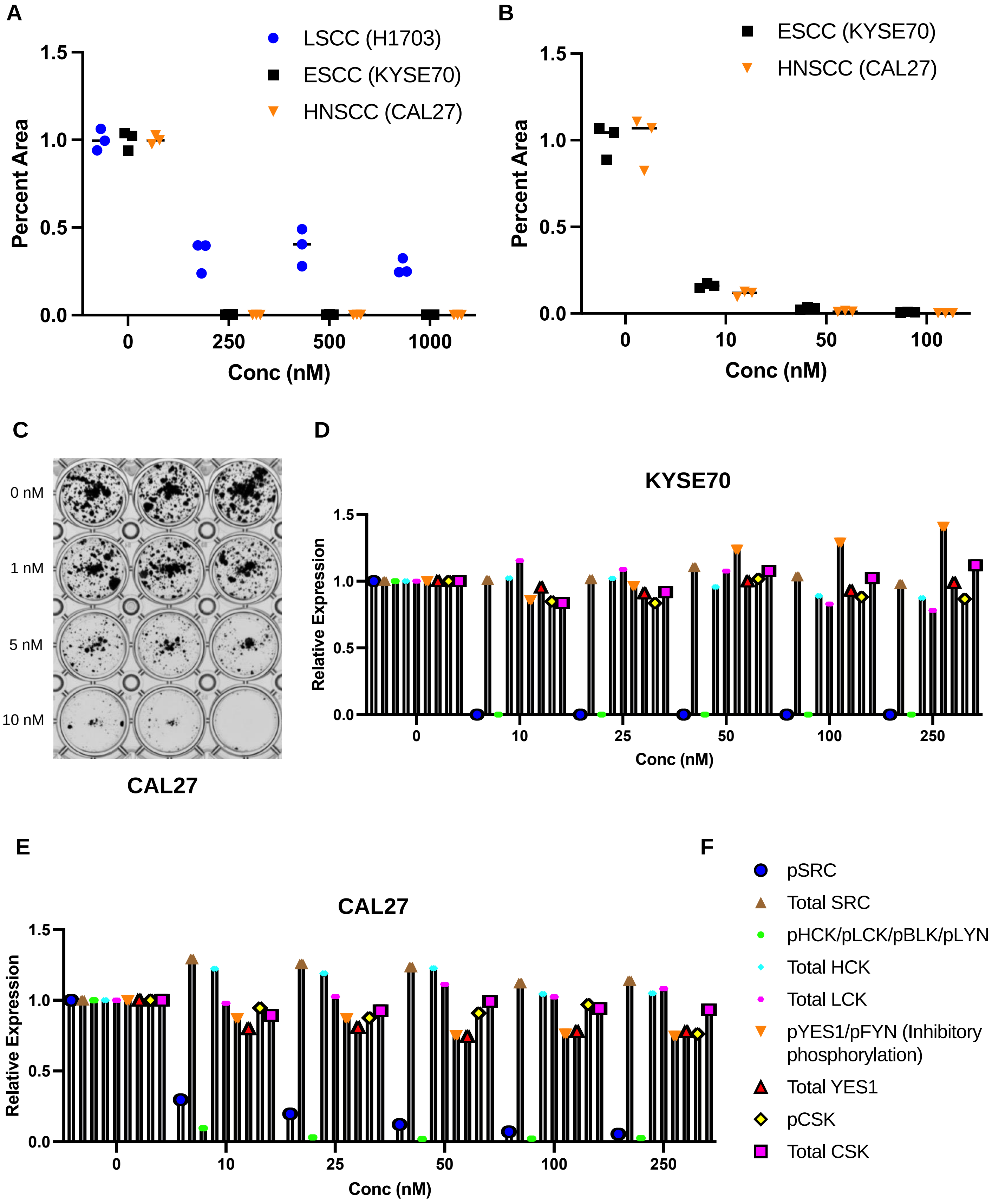

Supplementary Figure 1. ImageJ quantification of CFA and western blot data. ImageJ analysis of CFA at **A)** high and **B)** low concentrations of NXP900 **C)** Long-term treatment of CAL27 cells at very low concentrations of NXP900 (1nM, 5nM, 10nM) **D)** ImageJ analysis of western blots demonstrating the effect of NXP900 treatment on SFK and CSK activation in **D)** KYSE70 and **E)** CAL27 cell lines **F)** Legends for D and E

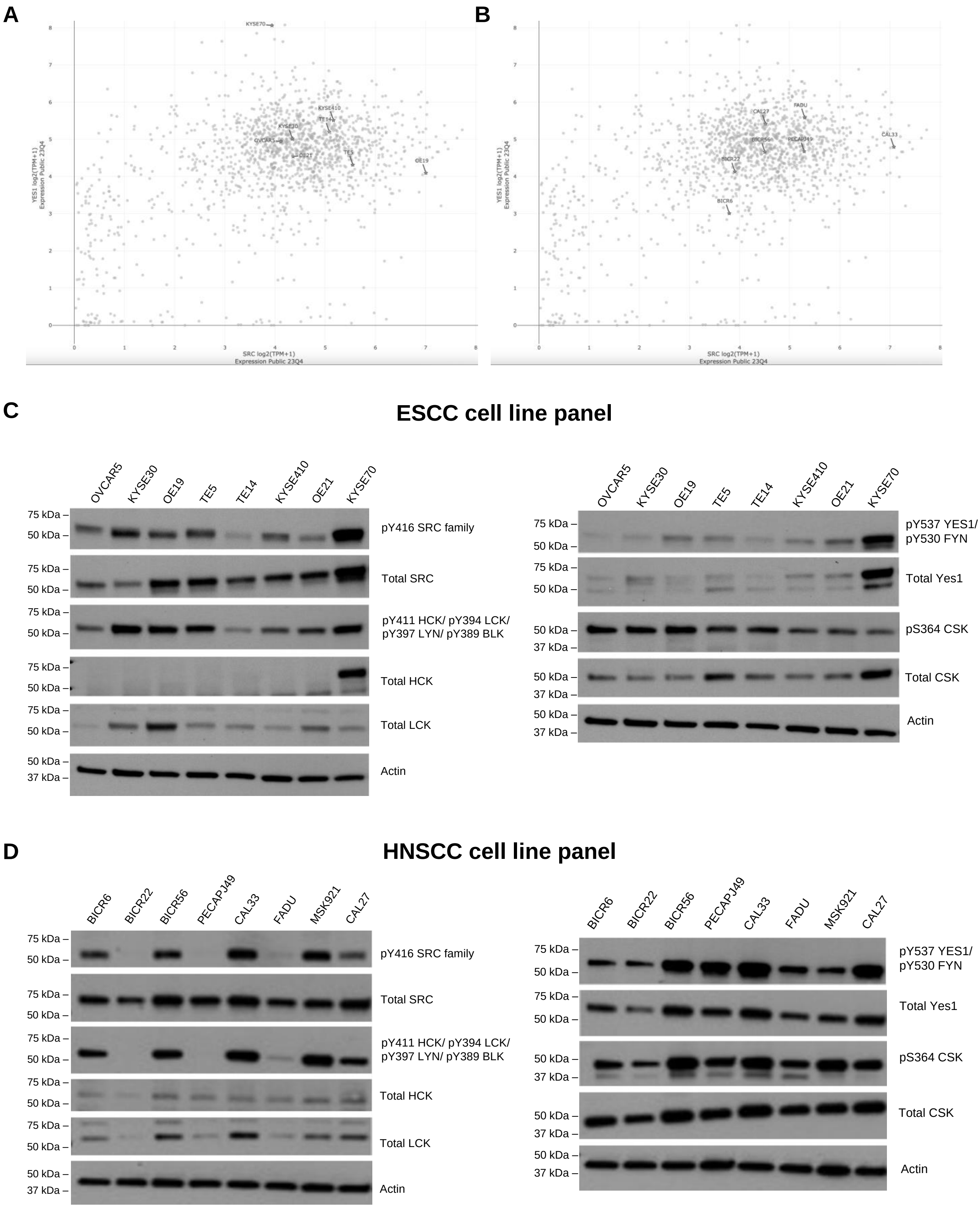

Supplementary Figure 2. Endogenous expression and activation levels of Src and Yes1 in ESCC and HNSCC cell line panel. A) and **B)** DepMap analysis of mRNA expression levels of *SRC* (x-axis) and *YES1* (y-axis) transcripts in A) ESCC and B) HNSCC cell line panel **C)** and **D)** Endogenous SFK activation and protein expression levels in C) ESCC and D) HNSCC cell line panel

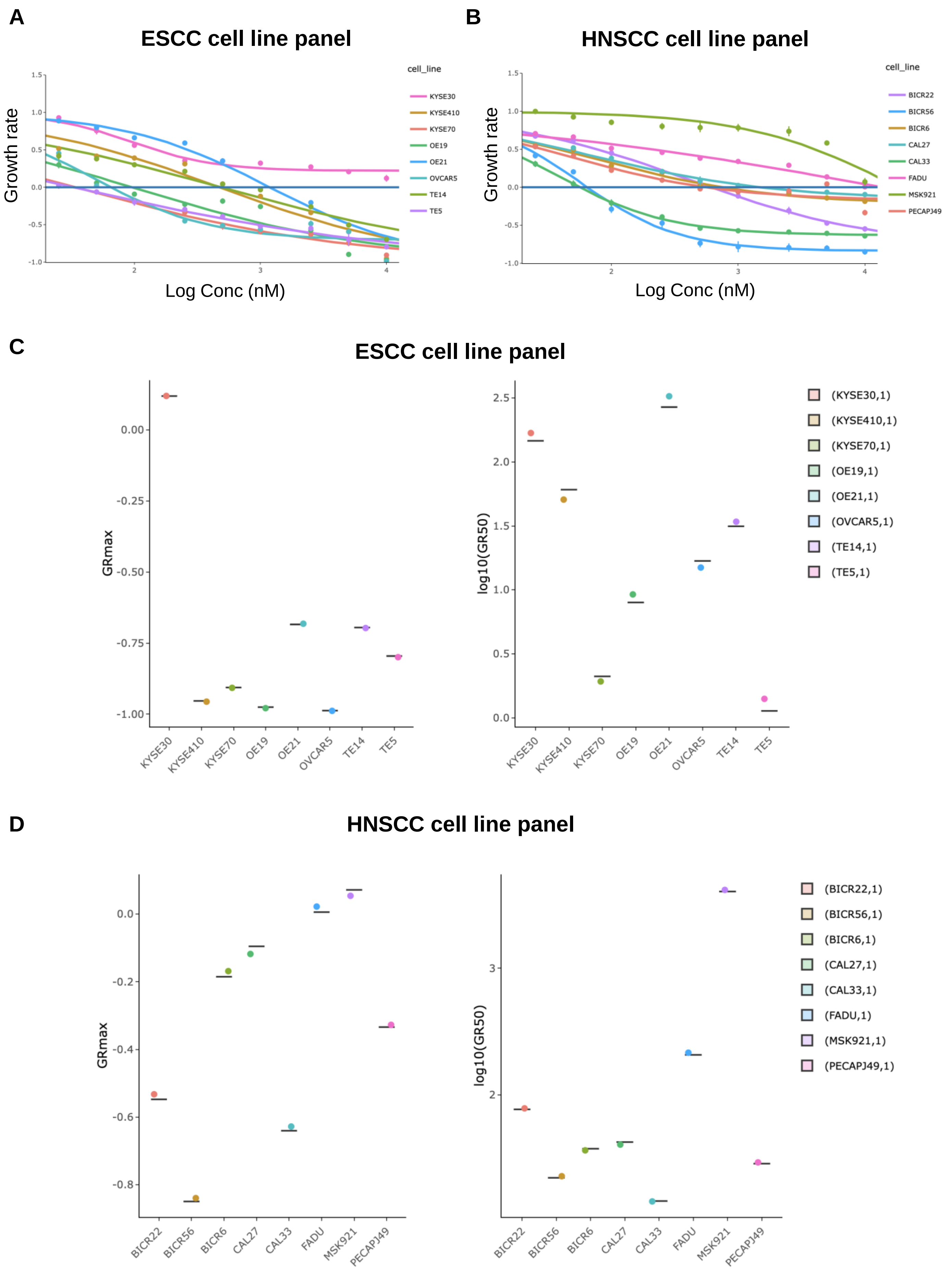

Supplementary Figure 3. GR analysis. Dose-response curve using GR metrics in **A)** ESCC and **B)** HNSCC cell line panel. GRmax and GR50 comparison across **C)** ESCC and **D)** HNSCC cell line panel using online GR calculator (http://www.grcalculator.org/grcalculator/)

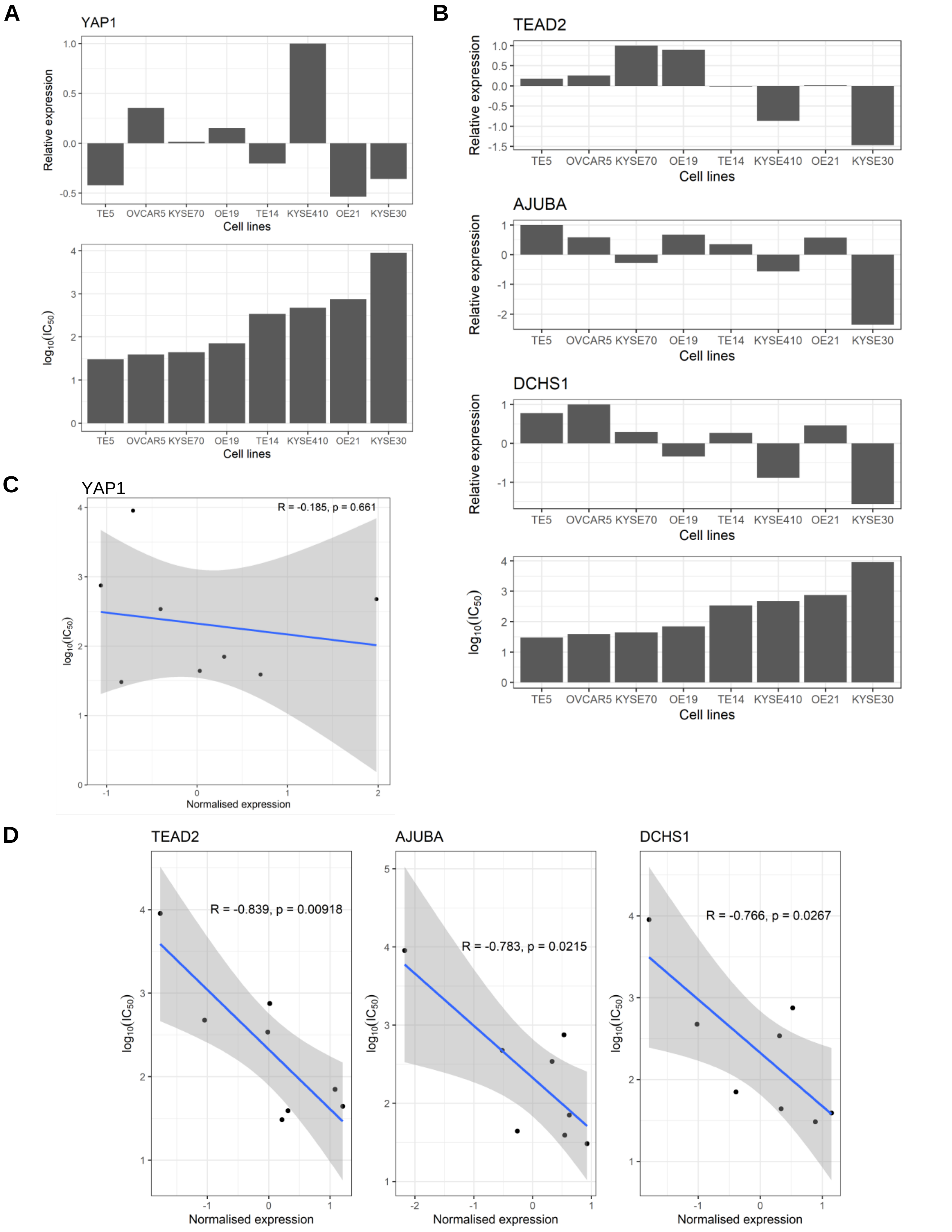

Supplementary Figure 4. TEAD2 expression correlates with NXP900 sensitivity in ESCC. A) and **B)** Relative mRNA expression levels and NXP900 sensitivity across ESCC cell line panel **C)** YAP1 mRNA expression and **D)** TEAD2, AJUBA and DCHS1 mRNA expression level was correlated with average $\log_{10}(IC_{50})$ using Pearson's correlation coefficient (R) in ESCC cell line panel. Blue line represents linear regression line with associated 95% confidence intervals shaded.
