# Supplementary material for: The SRC family kinase inhibitor NXP900 demonstrates potent antitumor activity in squamous cell carcinomas
Source: J Biol Chem. 2024 Jul 31;300(9):107615. doi: 10.1016/j.jbc.2024.107615 (PMC11388391; doi:10.1016/j.jbc.2024.107615)
Supplement: Supplementary Methods [file mmc2.docx]

**Bioinformatics analysis of expression of key hippo pathway members and correlation with NXP900 sensitivity across** **ESCC and HNSCC cell line panels**

RNAseq data obtained from the 22Q4 DepMap release from the Cancer Cell Line Encyclopedia (CCLE) was used to correlate mRNA expression of key hippo pathway members with sensitivity to NXP900 treatment across our ESCC and HNSCC panels. RNAseq data from DepMap was median-of-ratio normalized and a variance-stabilizing transformation applied using the ‘DESeq2’ package in R. Gene expression was scaled, centered and relative expression was calculated relative to the highest expression in the ESCC and HNSCC panels used in this study separately. IC50 values for NXP900 obtained from cell viability assays was averaged across two replicates for each cell line and the logarithm (base 10) of the average was taken for correlation with mRNA expression.
