# Supplementary material for: The SRC family kinase inhibitor NXP900 demonstrates potent antitumor activity in squamous cell carcinomas
Source: J Biol Chem. 2024 Jul 31;300(9):107615. doi: 10.1016/j.jbc.2024.107615 (PMC11388391; doi:10.1016/j.jbc.2024.107615)
Supplement: Supplementary Table [file mmc3.docx]

**Supplementary Table S1.** List of antibodies used in this study

| Antibody | Source | Identifer |
| --- | --- | --- |
| Phospho-Src Family (Tyr416) | Cell Signaling Technology | Cat #2101 |
| Src Antibody | Cell Signaling Technology | Cat #2108 |
| Phospho-LYN (Tyr397)/LCK (Tyr394)/HCK (Tyr411)/BLK (Tyr389) (E5L3D) Rabbit mAb | Cell Signaling Technology | Cat #70926 |
| Hck (E1I7F) Rabbit mAb | Cell Signaling Technology | Cat #14643 |
| Lck Antibody | Cell Signaling Technology | Cat #2752 |
| Csk (C74C1) Rabbit mAb | Cell Signaling Technology | Cat #4980 |
| Phospho-CSK (Ser364) Polyclonal Antibody | Invitrogen | Cat #PA5-40214 |
| Yes Antibody | Cell Signaling Technology | Cat #3201 |
| Recombinant Anti-Fyn (phospho Y530) + Yes1 (phospho Y537) antibody [EPR13512] | Abcam | Cat #ab188319 |
| YAP antibody | Abcam | Cat# ab52771 |
| Phalloidin-iFluor 594 secondary | Abcam | Cat# ab176757 |
| Alexa-Fluor 488 secondary | Therma Fisher Scientific | Cat# A-11008 |
